# Convolutional Autoencoding and Gaussian Mixture Clustering for Unsupervised Beat-to-Beat Heart Rate Estimation of Electrocardiograms from Wearable Sensors

**DOI:** 10.3390/s21217163

**Published:** 2021-10-28

**Authors:** Jun Zhong, Dong Hai, Jiaxin Cheng, Changzhe Jiao, Shuiping Gou, Yongfeng Liu, Hong Zhou, Wenliang Zhu

**Affiliations:** 1 Suzhou Institute of Biomedical Engineering and Technology, Chinese Academy of Sciences, Suzhou 215163, China; zhongj@sibet.ac.cn (J.Z.); haidong1029@163.com (D.H.); zhouh@sibet.ac.cn (H.Z.); wlzhu@sibet.ac.cn (W.Z.); 2 Suzhou Institute of Biomedical Engineering and Technology, University of Science and Technology of China, Hefei 230026, China; 3 Key Laboratory of Intelligent Perception and Image Understanding of Ministry of Education of China, School of Artificial Intelligence, Xidian University, Xi’an 710071, China; jxcheng@stu.xidian.edu.cn (J.C.); cjiao@mail.missouri.edu (C.J.); shpgou@mail.xidian.edu.cn (S.G.)

**Keywords:** autoencoding, electrocardiogram, Gaussian mixture clustering, heart rate estimation

## Abstract

Heart rate is one of the most important diagnostic bases for cardiovascular disease. This paper introduces a deep autoencoding strategy into feature extraction of electrocardiogram (ECG) signals, and proposes a beat-to-beat heart rate estimation method based on convolution autoencoding and Gaussian mixture clustering. The high-level heartbeat features were first extracted in an unsupervised manner by training the convolutional autoencoder network, and then the adaptive Gaussian mixture clustering was applied to detect the heartbeat locations from the extracted features, and calculated the beat-to-beat heart rate. Compared with the existing heartbeat classification/detection methods, the proposed unsupervised feature learning and heartbeat clustering method does not rely on accurate labeling of each heartbeat location, which could save a lot of time and effort in human annotations. Experimental results demonstrate that the proposed method maintains better accuracy and generalization ability compared with the existing ECG heart rate estimation methods and could be a robust long-time heart rate monitoring solution for wearable ECG devices.

## 1. Introduction

Based on data from the National Health and Nutrition Examination Survey (NHANES), 2013 to 2016, the prevalence of cardiovascular disease (CVD) in adults over 20 years of age is 48.0% overall in the US (121.5 million in 2016) and increases with age in both males and females. CVD prevalence, excluding hypertension, is 9.0% overall (24.3 million in 2016) [1]. Cardiovascular disease has also become the number one disease, with high incidence rates in the world [2]. Some researchers have proposed that it is necessary to carry out real-time monitoring for patients with basic heart disease [3]. If real-time monitoring of heart health can be carried out in daily life, the disease can be found as early as possible and be treated in time, which is more conducive to improving the living quality of patients [4]. Heart rate is one of the most important ways to evaluate health, especially for patients with cardiovascular disease. Heart rate variability, as an important indicator of health, is helpful in the early diagnosis of diseases [5]. Therefore, developing robust long-term heart monitoring devices and beat-to-beat heart rate estimation methods are of great importance.

**Figure 1:**
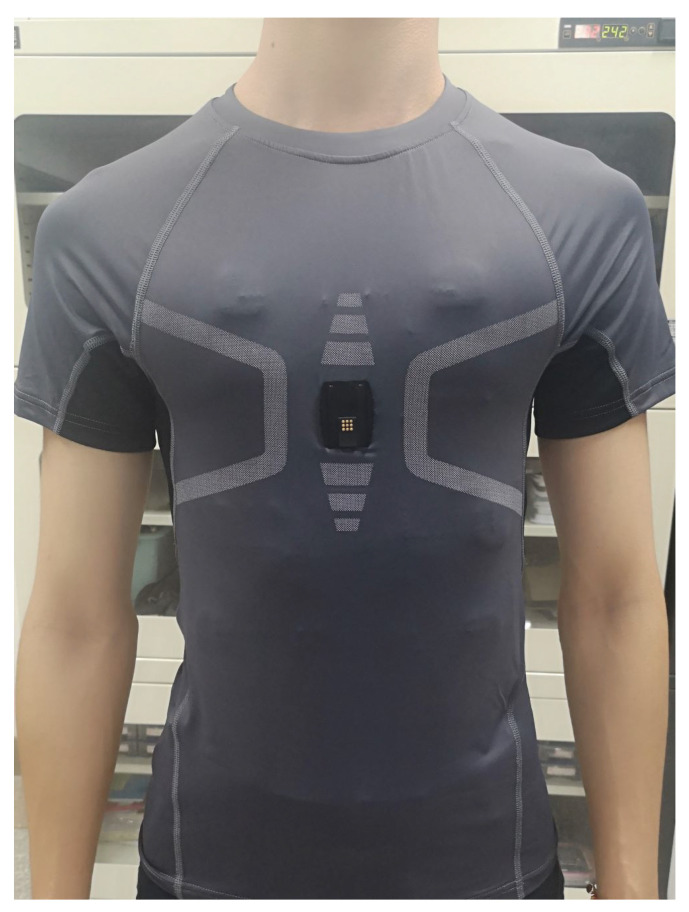
A wearable ECG device that collects the ECG signals through three embedded fabric electrodes in the vest.

An ECG is the most widely applied heart monitoring method in clinics. An ECG signal contains rich health information, reflecting vital signs. Through the analysis of an ECG signal, many indicators reflecting heart functions can be obtained, which is of great significance for subsequent diagnosis and treatment. ECG signals maintain the advantages of stability and accuracy, which are ideal for heart health analysis. However, a traditional ECG device requires electrodes to be attached to the patient’s chest, and requires professional operating, which is inconvenient for daily operation. At present, many wearable devices for heart rate measurements have been developed, such as chest belts and vests [6,7,8,9,10,11]. These wearable devices are comfortable to wear; they are easy for operation, at any time or for long-term monitoring. These wearable smart devices record ECG, respiration, body temperature, and other related physiological parameters, which are more conducive to the diagnosis of diseases. For patients, it can better achieve a real-time analysis of the disease, with instant preventive treatment; for healthy people, it can monitor and record the relevant heart condition changes during exercise, and can carry out health monitoring on various indicators of the body in daily life. Figure 1 shows an ECG embedded vest developed by the Suzhou Institute of Biomedical Engineering and Technology, Chinese Academy of Sciences, which can collect human ECG signals in real time. The vest records ECG signals through three embedded fabric ECG electrodes, which can be comfortably worn in daily life.

For the signals collected by these wearable devices, because they do not directly contact the skin, the signal quality of these electrodes is not as good as that measured by the medical electrocardiograph. In addition to the baseline signal drift and hardware frequency interference, subjects may inevitably have noise interference caused by sudden moving or even moving in the long-term, which cast challenges to signal analysis from the wearable devices.

Heart rate estimation from ECG data using supervised machine learning methods typically require adequate amounts of training data, with accurate heartbeat annotations. However, obtaining such volumes of labeled data could cost tremendous time and effort, and is sometimes infeasible. For example, accurately labeling each cardiac cycle may require high medical expertise and dedicated attention from the medical analyst. Ground truth obtained from other intrusive measurements, such as a finger sensor, may bring intrinsic inaccuracy to the ground truth when the signal quality is low, e.g., timing misalignment between the two sensor systems. Heart rate estimation using frequency domain analysis maintains high efficiency and does not need labeled training data; however, these methods usually estimate a heart rate over a period of a signal and hardly detect heartbeats. Thus, developing an unsupervised beat-to-beat ECG heart rate estimation method that does not rely on accurate locations of each heartbeat is more desirable and practical for daily heart monitoring using wearable ECG sensors.

In this paper, the autoencoding technology is introduced into the feature extraction of an ECG signal, and a beat-to-beat heart rate estimation based on Gaussian mixture clustering is proposed for ECG signals from wearable sensors. Extending our previous study [12], in order to further reduce the dependence on manual labeling, we introduce the autoencoder Gaussian mixture model (AE-GMM) into an ECG heart rate estimation. The deep one-dimensional convolutional autoencoder network is trained to extract ECG signal heartbeat features by minimizing the reconstruction error; then, the adaptive number of categories is introduced into Gaussian mixture clustering for heartbeat detection and heart rate estimation. Compared with the existing ECG heart rate estimation methods, the contributions of the proposed work are:

(1) As an unsupervised learning method, this method does not require precise manual marking of the heartbeat locations, which can save labor costs, time, and is more practical. Compared with the traditional signal processing methods, this method relies on the powerful representation ability of the convolutional neural network to significantly improve the accuracy of heart rate estimation. Its advantage lies in the fusion of three-dimensional signals into one-dimensional features through the autoencoder, which can make full use of the information of the three-lead ECG signals in an unsupervised manner to obtain more prominent features.

(2) The collection of ECG signals is non-invasive through a wearable device, which makes the collected signals inevitably vulnerable to interference and noise. Among them are the distortion of the signal caused by motion and noise. The previous signal processing methods are difficult to achieve satisfactory estimation performance on such noisy signals, so we combined the deep autoencoder with Gaussian mixture modeling to propose this unsupervised learning method to adaptively model the high-level heartbeat features and achieve robust beat-to-beat heart rate estimation.

(3) The proposed method was fully verified on the recently developed variable ECG sensors by the Suzhou Institute of Biomedical Engineering and Technology, Chinese Academy of Sciences, providing a reliable beat-to-beat heart rate estimation solution for wearable devices.

## 2. Related Work

**Figure 2:**
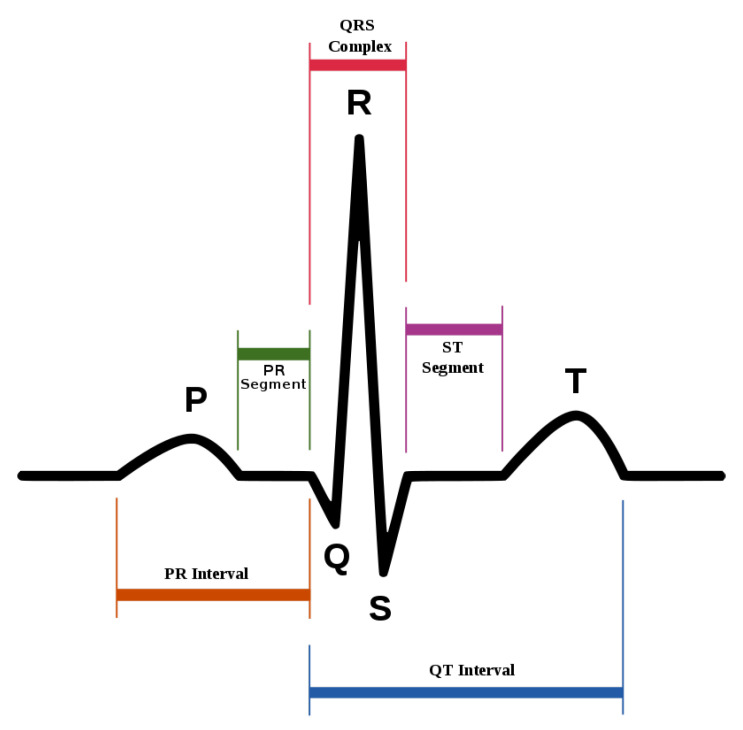
The standard waveform of ECG signal.

ECG heartbeat waveforms maintain certain discriminative features that are instrumental for heart rate analysis, among which, the most important one is the detection of QRS complex, namely, the R wave shown in Figure 2. However, in practice, the mixture of system noise and/or body movements in ECG waveform make it quite challenging for advanced analysis in the long-term. A number of effective methods have been proposed in the literature. Table 1 lists some of the related methods, and we divide them into three categories. W. J. Tompkins proposed the Pan–Tompkins method, which is the most classic R-wave detection method based on thresholding [13]. The Pan–Tompkins method consists of two parts: the signal preprocessing stage and R-wave detection in the decision-making stage, maintaining high real-time performance and high detection accuracy, while also reducing the false detection of T-waves in the ECG recordings. Ramakrishnan et al. combined adaptive moving window difference thresholding and front back difference thresholding to detect the R wave [14]. Banerjee et al. applied discrete wavelet transform to learn frequency features and then conducted classification of anteroseptal myocardial infarction [15]. Minami et al. designed a Fourier transform neural network for real-time detection of ventricular tachyarrhythmia [16]. Sun et al. applied the morphological transform to learn a discriminative template, then detected fiducial points in an ECG signal [17]. Kim et al. applied the difference operation method for R wave detection for a patch-type ECG remote monitoring system [18]. Dewangan et al. combined wavelet and morphological coefficients as input features and applied the neural network for arrhythmia classification [19]. Osowski et al. extracted the high-order statistics and Hermitian features of the QRS complex and used a support vector machine for ECG heartbeat classification [20]. Other classic ECG heart rate estimation methods include filter bank [21], phase space [22], the QRS multilevel Teager energy operator (MTEO) [23], automatic multiscale-based peak detection (AMPD) [24], and the UNSW QRS detection algorithm [25]. The filter bank [21] method combines several band-pass filters in order to better delineate the QRS complexes. The phase space [22] method adopts nonlinear phase space reconstruction recorded by ECG to identify QRS complexes. MTEO [23] uses the multilevel Teager energy operator to locate the QRS complex. UNSW QRS detection [25] applies QRS detection in the ECG signal collected in the telehealth environment. The proposed method combined the filtered ECG amplitude and derivative as input features and an adaptive thresholding was applied, achieving more robustness for telehealth applications.

Traditional classification methods based on wavelet, morphological features, adaptive thresholding, and others rely too much on feature engineering. The accuracy of classification is limited by prior knowledge and often requires a lot of artificial features to assist analysis, which is complex and time-consuming in practice. With the development of the deep learning theory and the increasing power of parallel computing, end-to-end deep learning based classification/regression methods emerge rapidly in ECG signal analysis. Oh et al. introduced the one-dimensional convolutional neural network (CNN) and long short-term memory (LSTM) for feature extraction and arrhythmia diagnosis from variable length ECG signals [26]. Fotiadou et al. proposed to use convolutional neural network and the LSTM network, which can fuse spatial and temporal information from multi-channel fetal ECG signals to directly predict fetal heart rate [27]. Lee et al. proposed an algorithm for detecting fetal QRS complexes in non-invasive fetal ECG (NI-FECG) signals based on convolutional neural networks, which can reliably detect fetal QRS complexes without separating the maternal ECG signals [28]. Because the NI-FECG signal contains a maternal ECG signal with a larger amplitude than the fetal ECG signal, it can be seen from the side that the deep network has a strong feature characterization ability.

Deep learning methods often require adequate amounts of accurately labeled data for training, which is usually very time- (and effort) consuming, and sometimes infeasible. Therefore, it is more appealing to develop unsupervised feature learning algorithms for ECG signal analysis. In [29], the K-singular value decomposition (K-SVD) method was adopted for sparse large ECG data feature extraction and QRS classification. Ref. [30] proposed a ECG heartbeat clustering method based on the unsupervised extreme learning machine and decision rule. Ref. [31] proposed a heartbeat detection method based on adaptive thresholding on the two-dimensional representation of ECG features obtained from a number of independent detection methods.

**Table 1:** The related works of R wave detection.

Algorithm Category	Year	Method
Traditional detection methods	1985	Pan–Tompkins [13]
	1999	Fourier-transform neural network [16]
	1999	Filter banks [21]
	2002	Phase space [22]
	2004	Support vector machine-based expert system [20]
	2005	Morphological transform [17]
	2010	Discrete wavelet transform [15]
	2011	Difference operation [18]
	2015	Multilevel Teager energy operator (METO) [23]
	2016	UNSW [25]
	2016	Discrete wavelet transform and artificial neural network [19]
	2017	Adaptive threshold [14]
Supervised deep neural network methods	2018	Combination of 1D-CNN and LSTM [26]
	2018	Convolutional neural networks [28]
	2020	Deep convolutional LSTM regression [27]
Unsupervised machine learning methods	2012	Clustering and multimethod approach [31]
	2016	Advanced K-means clustering algorithm and K-SVD [29]
	2016	Unsupervised ELM and decision rule [30]

## 3. Proposed Method

The goal of the proposed autoencoding Gaussian mixture model (AE-GMM) is to estimate the locations of each heartbeat and compute the heart rate of a subject through the ECG signal acquired by the wearable ECG device. Specifically, let $X=\{x_{1},x_{2},\ldots ,x_{n}\}$ denote the input ECG data, $\hat{X}$ denote the reconstructing ECG data and $Y=\{y_{1},y_{2},\ldots ,y_{n}\}$ denote the hidden layer features extract from the encoder-decoder.

### 3.1. The 1D Convolutional Self-Encoder

**Figure 3:**
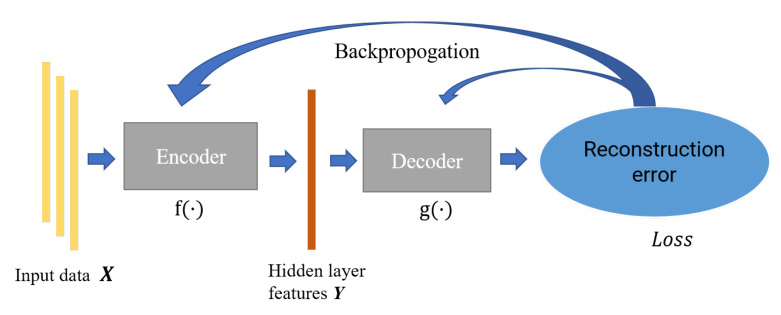
The structure of autoencoder.

Autoencoder is a feature extraction model composed of the neural network, which consists of an encoder and decoder. The encoder is mainly responsible for the feature representation of data, while the decoder is responsible for reconstructing the original data through the characterization results; the parameters of the encoder–decoder are optimized by minimizing the reconstruction error. In this paper, we constructed an incomplete one-dimensional convolutional autoencoder by limiting the output dimension of the encoder to be smaller than the original data. As shown in Figure 3, we used the incomplete learning mechanism to constrain the autoencoder to capture the most significant features of the ECG data.

For the input data x, the encoding process that maps it from the input layer to the hidden layer can be represented as follows:

(1) $$Y=f\left(W_{en}\cdot X+b_{en}\right),$$

where $W_{en}$ and $b_{en}$, respectively, are the connection weights and biases of the coding module, Y is the encoded hidden layer features, and f(·) is the coding mapping function of the input data.

The decoding process of reconstructing training samples $\hat{X}$ from the hidden layer can be expressed as follows:

(2) $$\hat{X}=g\left(W_{de}\cdot Y+b_{de}\right),$$

where $W_{de}$ and $b_{de}$, respectively, are the connection weights and offsets of the decoding module, and g(•) is the decoding mapping function of the encoding feature.

The training process can be expressed as minimizing the following loss function,

(3) $$\min_{\theta }\;Loss\left(X,\hat{X},\theta \right)={\left(X-\hat{X}\right)}^{2}.$$

In Equation (3), Loss denotes the loss function, which is the mean square error in this paper, and θ is the training parameters for the network.

(4) $$ReLU\left(x\right)=\left\{\begin{array}{c} 0\;x\leq 0\\ x\;x>0 \end{array}\right.$$

The activation function used in this paper is Rectified Linear Unit (ReLU), and its formula is shown in Equation (4). The activation function not only introduces nonlinear factors into the network to improve the feature extraction ability of the model, it also effectively avoids the gradient disappearance.

### 3.2. Gaussian Mixture Clustering

Gaussian mixture clustering is a probabilistic model that infers the mixture of a finite number of Gaussian distributions generating the observed data. In theory, the Gaussian mixture model can represent any complex distribution. In this paper, we use the Gaussian mixture distribution to model the features extracted from the autoencoder for each ECG heartbeat cycle.

For the multiple Gaussian distribution used to model the features Y, let p(Y∣μ,Σ) denote the probability density of the data, then the Gaussian mixture distribution composed of *K* mixture components can be defined as,

(5) $$p_{M}=\sum_{k=1}^{K}\alpha_{k}p\left(Y|\mu_{k},\Sigma_{k}\right)$$

In Equation (5), $p\left(Y|\mu_{k},\Sigma_{k}\right)$ is Gaussian distribution density, and $\mu_{k}$, $\Sigma_{k}$ are the mean vector and covariance matrix of the $k^{th}$ Gaussian mixture component, respectively. $\alpha_{k}$ is the mixing coefficient of the $k^{th}$ Gaussian mixture, which satisfies:

(6) $$\alpha_{k}\in \left(0,1\right],\sum_{k=1}^{K}\alpha_{k}=1$$

Assuming feature $y_{i}$ follows a certain Gaussian mixture, let *Z* be the label of the Gaussian mixture component that generates the random variable $y_{i}$. The prior probability of *Z* is $p\left(Z=k\right)=\alpha_{k}$ and the posterior distribution is shown as Equation (7).

(7) $$\begin{array}{cc} p_{M}\left(Z=k|y_{i}\right) & =\frac{p\left(Z=k\right)p_{M}\left(y_{i}|Z=k\right)}{p_{M}\left(y_{i}\right)}\\ \; & =\frac{\alpha_{k}p\left(Y|\mu_{k},\Sigma_{k}\right)}{\sum_{k=1}^{K}\alpha_{k}p\left(Y|\mu_{k},\Sigma_{k}\right)} \end{array}$$

Assuming that the Gaussian mixture distribution of Y is known, Gaussian mixture clustering can divide the feature set into *K* clusters according to different Gaussian mixture components. The clustering result of $y_{i}$ can be expressed as:

(8) $$C_{y_{i}}=\arg\max_{k}p_{M}\left(Z=k|y_{i}\right)$$

For the features set $Y=\{y_{1},y_{2},\ldots ,y_{N}\}$, the parameter learning of Gaussian mixture distribution can be solved by expectation maximization (EM) algorithm [32]. Denoting the number of Gaussian mixture components in the sample to be *K*, the optimization process can be shown as follows:

(1) Initialize the model parameters of each Gaussian mixture component;
(2) Calculate the posterior probability $\gamma_{ik}=p_{M}$ of each feature $y_{i}$ generated by each mixed component according to Equation (7);
(3) Calculate the new mean vector μ of each Gaussian mixture component,
  (9) $$\mu_{k}^{\prime }=\frac{\sum_{i=1}^{N}\gamma_{ik}y_{i}}{\sum_{i=1}^{N}\gamma_{ik}},\;k=1,2,3,\ldots ,K$$
  and the new covariance matrix
  (10) $$\Sigma_{k}^{\prime }=\frac{\sum_{i=1}^{N}\gamma_{ik}\left(y_{i}-\mu_{k}^{\prime }\right){\left(y_{i}-\mu_{k}^{\prime }\right)}^{T}}{\sum_{i=1}^{N}\gamma_{ik}y_{i}},\;k=1,2,3,\ldots ,K$$
  and the new mixing coefficient
  (11) $$\alpha_{k}^{\prime }=\frac{\sum_{i=1}^{N}\gamma_{ik}}{N},\;k=1,2,3,\ldots ,K$$
(4) Update the parameters of each Gaussian mixture component;
(5) If the termination condition is satisfied, the final clustering classification is determined according to Equation (8). If not, repeat Step (2)∼(4) until the termination condition is satisfied.

### 3.3. Cluster Evaluation Function

In the proposed method, the Calinski–Harabasz index [33] was adopted as cluster evaluation index. The Calinski–Harabasz clustering evaluation function is used to calculate the scores of the clustering results under each category, and determine the heartbeat cluster for heartbeat confirmation.

For clusters, the Calinski–Harabasz score is expressed as the ratio of the within-cluster dispersion and the between-cluster dispersion. Higher Calinski–Harabasz scores are related to models with better defined clusters. The definition of Calinski–Harabasz index is shown as follows:

(12) $$s\left(k\right)=\frac{\mathrm{tr}\left(B_{k}\right)\left(N-K\right)}{\mathrm{tr}\left(W_{k}\right)\left(K-1\right)},$$

where *N* is the number of sample sets and *K* is the number of categories; tr(·) is the trace of the matrix, $W_{k}$ is the covariance matrix within the category, and $B_{k}$ is the covariance matrix between classes shown as follow:

(13) $$B_{k}=\sum_{k}n_{k}\left(c_{k}-c\right){\left(c_{k}-c\right)}^{T},$$

where $c_{k}$ is the center of the $k^{th}$ cluster, *c* is the center of the whole feature set and $n_{k}$ is the number of samples of the $k^{th}$ cluster.

It can be seen from the definition that the higher the Calinski–Harabasz index, the better the clustering performance (dense and well separated). The heartbeat cluster is confirmed by selecting the cluster with the highest Calinski–Harabasz score. As an unsupervised performance evaluation index, it is very suitable for heartbeat cluster confirmation for the proposed method in practice because of its low computational complexity and relatively stable performance.

### 3.4. Algorithm Model

The proposed AE-GMM model includes three parts: heartbeat feature extraction, heartbeat feature clustering and heartbeat cluster confirmation, and beat-to-beat heart rate calculation, which are illustrated in the following parts and summarized in Algorithm 1.
**Algorithm 1:**AE-GMM**01** Find all peak locations of the three lead ECG signals.**02** Extract data segment centered at each peak with radius *r* = 45 as the input feature for this candidate peak, resulting in feature tensor X in dimension [M, 91, 3], where M is the total number of peaks, 2*r* + 1 = 91 is the total length of th input heartbeat signals.**03** Standardize the input feature tensor *X*.**04** Train the autoencoding network and extract the compressed feature tensor *Y* with dimension (M, 91, 1).**05** Perform Gaussian mixture clustering as described in Section 3.2.**06** Determine the heartbeat cluster according to the Calinski–Harabasz clustering scores.**07** Calculate the beat-to-beat heart rate based on the R wave locations.

**Figure 4:**
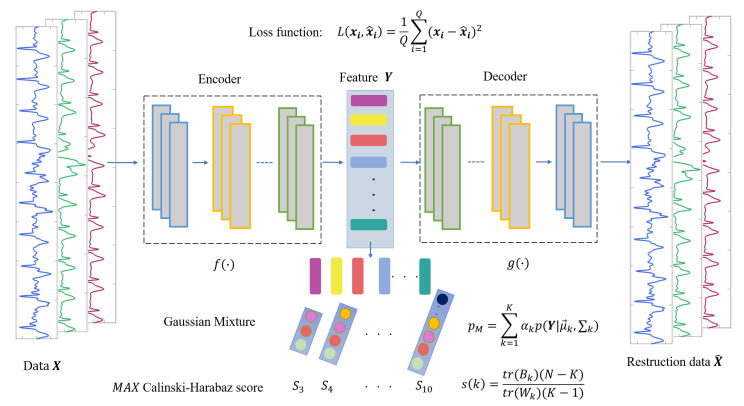
The structure of AE-GMM, which contains encoder and decoder convolution layers and Gaussian mixture clustering.

**Figure 5:**
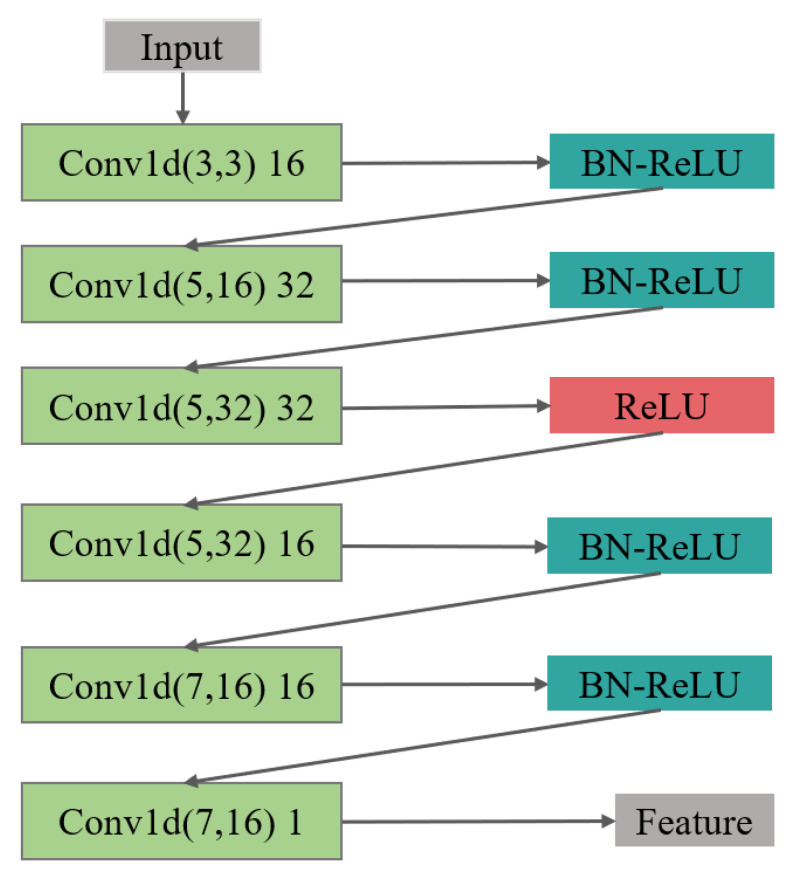
The feature extraction structure of the encoder is composed of 6 layers of 1D CNN, batch normalization (BN), and ReLU activation layer.

(1) In the feature extraction section, the encoding and decoding parts of the convolutional autoencoder are the one-dimensional convolutional and deconvolution network, respectively. The structure of the model is shown in Figure 4, in which, the structure of the convolutional submodule is shown in Figure 5. Through the operation of the convolution kernel, as shown in Figure 5, the three-lead input signal is merged into one-dimensional, with length 91 samples.

(14) $$L\left(x,\hat{x}\right)=\frac{1}{Q}\sum_{i=1}^{Q}{\left(x_{i}-\hat{x_{i}}\right)}^{2}$$

The loss function is shown in Equation (14), where $x_{i}$ and $\hat{x_{i}}$ are the input and output of the one-dimensional convolutional autoencoder, respectively, and *Q* is the batch size.

**Figure 6:**
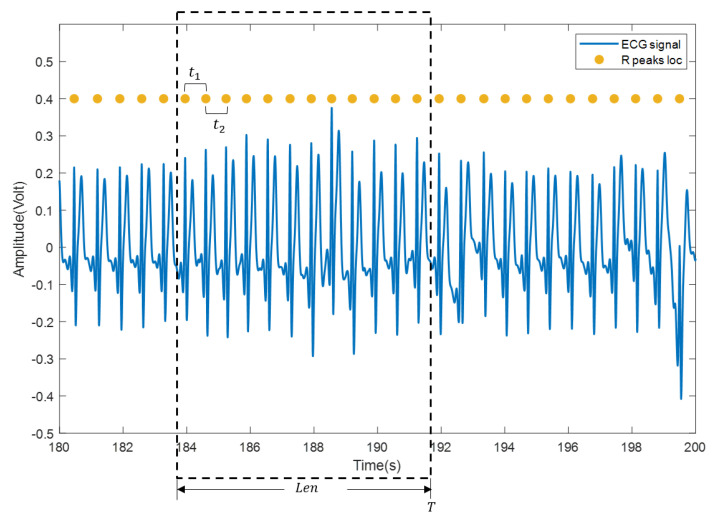
Calculation of beat-to-beat heart rate. The blue curve is the ECG signal, and the yellow solid dot is the reference position of the R wave.

(2) In the Gaussian mixture clustering section, we first set the initial number of categories in the range 3 ∼ 10, then traverse the range as the number of clusters for Gaussian mixture clustering and obtain the corresponding clustering results. In the end, the Calinski–Harabasz score of each clustering result is computed in the clustering evaluation step; the clustering result with the highest score is determined as the heartbeat cluster.

(3) The heartbeat locations are confirmed according to the heartbeat cluster and the beat-to-beat heart rate is calculated. Specifically, mean time interval of the confirmed heartbeats within a sliding window (with length Len, usually set to 30 s or 60 s) is firstly computed and then transformed into beat/min, as shown in Figure 6. Suppose that there are (m+1) heartbeats in the sliding window and the time interval between every two adjacent heartbeats in the sliding window is $\left\{t_{1},t_{2},\cdots ,t_{m}\right\}$, the beat-to-beat heart rate(hr) at time *T* is calculated as:

(15) $$hr\left(T\right)=\frac{60}{\mathrm{mean}\left\{t_{1},t_{2},\ldots ,t_{m}\}\right.}$$

**Figure 7:**
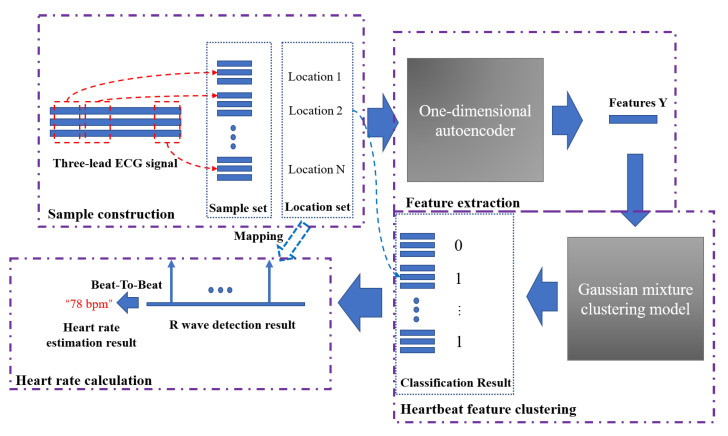
The complete heart rate estimation process of the AE-GMM method.

Figure 7 shows the entire heart rate estimation process of the algorithm AE-GMM. The purple dashed boxes in the figure represent the four key parts of the heart rate estimation process. The first part is the sample construction. This process performs data normalization, signal peak acquisition, signal interception, etc., as described in Algorithm 1. After the autoencoder training is completed, the encoder in the autoencoder will be used to extract the features of the samples, as shown in Figure 4. Then input the extracted features into the Gaussian mixture clustering model to get the clustering result of the sample, as shown in Figure 7, “0” in the classification result indicates that the sample is not a heartbeat, and “1” indicates a true heartbeat. Finally, the heartbeat sequence diagram can be obtained by combining the samples classified into the heartbeat and the position information of the samples, and then the heart rate can be easily calculated, according to Figure 6 and Equation (15).

## 4. Experiment

The study was conducted according to the guidelines of the Declaration of Helsinki, and approved by the Institutional Review Board, the Suzhou Institute of Biomedical Engineering and Technology, Chinese Academy of Sciences.The proposed method was evaluated on the ECG signal individually collected from healthy subjects by the wearable ECG sensors. Informed consent was obtained from all subjects involved in the study. In the training phase, the learning rate was 0.0005, the batch size was 128, and the Adam optimizer was adopted for optimization. Since the proposed method was unsupervised and no manually labeled information was required in the modeling process, it was not necessary to divide the data into the training set and the test set. The training process stops when the loss is less than a preset small threshold or the number of iterations is greater than 1500 epochs. In the clustering section, the initial number of categories ranges from 3 to 10. The conditions for clustering to stop is that the number of iterations is greater than 100 or the parameter update amount is less than 0.001. The experiments were conducted on a workstation with Intel i7 9700k CPU and NVIDIA 2080Ti GPU with 11 GB memory, under Python3.6 and a TensorFlow 1.4 environment.

### 4.1. Data Preprocessing

Ten healthy subjects were selected to participate in the evaluation and the heart signals were collected by the wearable ECG sensors shown in Figure 1. Each subject was required to collect ECG signals for at least 3 min in a sitting state. The signal from each subject was a three-channel lead data sampled at 100 Hz, and the data length is shown in Table 2.

Due to the ECG signals containing noise, such as breathing, body movement, gastrointestinal movement, etc., a second-order Butterworth filter with a lower cut-off frequency of 0.4 Hz and upper cut-off frequency of 10 Hz was applied for noise removal.

**Figure 8:**
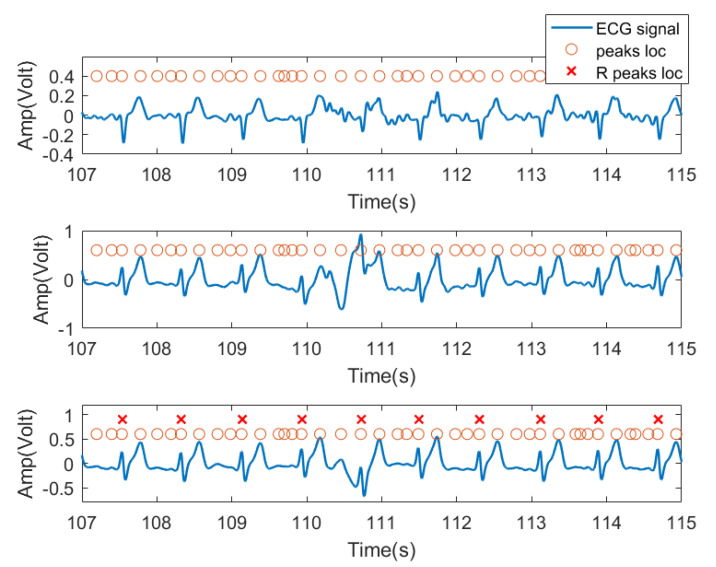
Data segment diagram. The blue curve is the ECG three-lead signal, and the orange circle represents the position of each signal segment intercepted by the point at the center with the left and right radius r, and the red X are the ground truth of R wave locations.

In this experiment, we used the time domain segments after filtering from received ECG signals as input features. Specifically, for each subject, because the peak position of each lead signal cannot be a one-to-one correspondence, the peak locations from one lead signal were labeled as the peak location of the entire ECG signal received by three channels as candidate R peak locations (possible heartbeat locations). As shown in Figure 8, the third row of signals was chosen as the reference signal. For each peak, we extracted a data segment centered at each peak with radius *r* as the training feature for this peak location. In practice, *r* was set to 45, resulting in 91 sample long features (corresponding to 0.91 s signal, 45 samples before and after the peak). This setting was verified in our previous work [34] and was found to be the typical length of a heartbeat pattern. Figure 8 shows the three lead ECG signals from subject no. 2, as an example, where filtered ECG signals collected by three ECG transducers were plotted. The orange circles denote the signal peaks, and the red **X** denote the example locations of R waves manually labeled by us and confirmed by a medical expert. The peak of the R wave in the ECG signal is the most prominent feature. Based on this, we assume that the heartbeat will only appear at the peak position.

In order to reduce the variance between input features, all input segments are standardized to have zero mean and unit variance.

### 4.2. Analysis of Experimental Results

In this section, we used a 30s sliding window to calculate beat-to-beat heart rates. The mean absolute error between the calculated heart rate and the real heart rate was calculated. The comparison methods, as Table 3 shows, include four unsupervised traditional QRS wave monitoring methods for ECG signals provided by an open MATLAB toolbox [35], which are: PT [13], AMPD [24], MTEO [23], UNSW [25]; ensemble learning methods, XGBoost [36], which have achieved good performance in many bioinformatics analysis; Hilbert Transform (HT) [37]; and Evo-MIACE [12]. Specifically, HT and Evo-MIACE are non-invasive heart rate estimation methods for the ballistocardiograms proposed by our collaborators, which we believe could be good comparisons and provide insightful evaluation on an ECG signal. For the HT method [37], the collected signal is decomposed into the heartbeat component and the harmonic component in the frequency domain by Hilbert transform; the heartbeat frequency component is used to compute heart rate. Since HT requires a period of a signal for frequency analysis, the heart rate is estimated through every 15-second long signal. Evo-MIACE [12] is a weakly supervised method that combines evolutionary optimization and multiple instance learning to learn a heartbeat “concept”. Then the heartbeat concept is used as a template to match the ECG signal and detect heartbeat.

**Figure 9:**
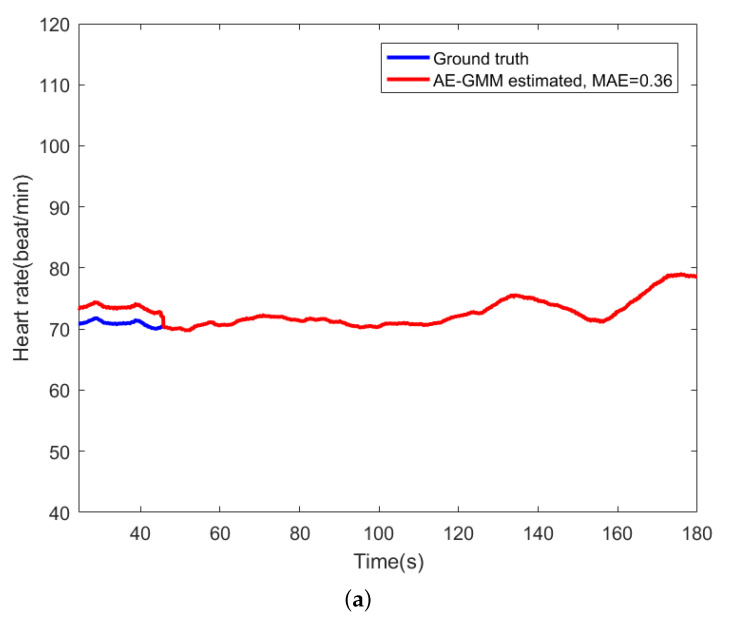

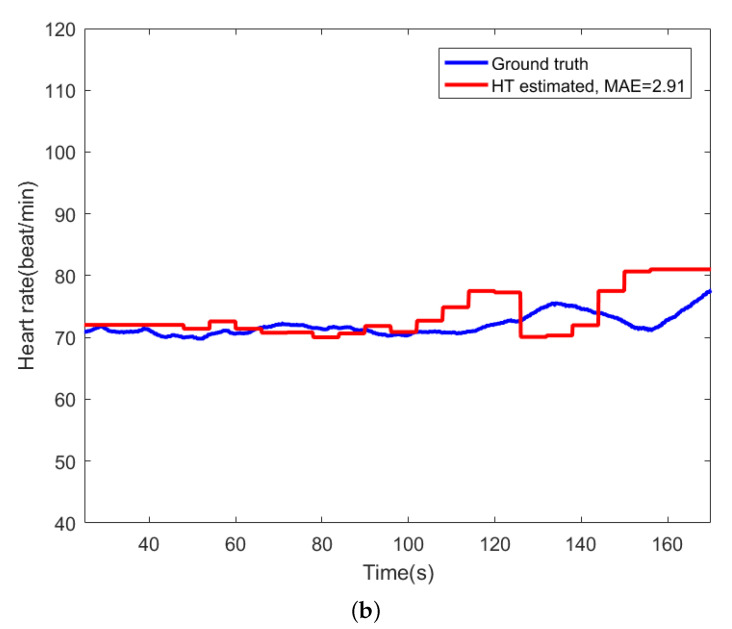
Comparison of heart rate estimation between AE-GMM and HT on subject no. 10. (**a**) Heart rate estimation of AE-GMM on subject no. 10. (**b**) Heart rate estimation of HT on subject no. 10.

For comparison, Figure 9 shows the beat-to-beat heart rate estimated by the proposed AE-GMM method and HT from subject no. 10. Figure 9a shows the beat-to-beat heart rate estimated by the proposed AE-GMM method from subject no. 10, where one can clearly see that the real time estimation of the heart rate, by the proposed methods, is very stable and accurate; expect an overestimate at the beginning. Figure 9b displays the heart rate estimated by the HT method, which is limited by the 15 s segment and not suitable for real time beat-to-beat heart rate estimation.

**Figure 10:**
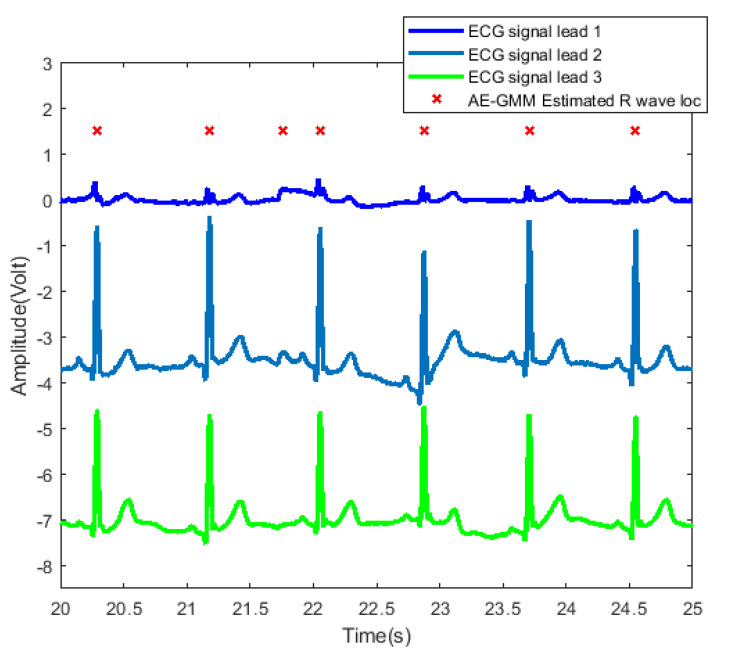
Heartbeat detection result of AE-GMM on subject no. 10. The figure shows the signals of three leads from 20 to 25 s, and the horizontal axis of leads 2 and 3 are shifted downward by 3.5 and 7, respectively.

In order to further examine and analyze the results, Figure 10 shows the detailed heartbeats confirmed by the proposed method. From Figure 10, one can clearly see that there exists only one false alarm heartbeat, where a non-R wave position is detected as an R wave at about 21.8 s. Since the sliding window size is 30 s, the influence of the overestimate of the heart rate lasts from 21.8 s to 51.8 s.

Table 4 summarizes the heart rate estimation error of AE-GMM and the comparison algorithms evaluated individually on the ten subjects. The proposed AE-GMM method achieved the overall best heart rate estimation performance, with average error smaller than 1 beat/min. It can be seen from Table 4 that some typical ECG heart rate estimation methods achieved excellent performance on several subjects, but failed (providing very large estimation error) for wearable ECG lead signals with relatively poor signal quality. Some methods detect Q, P, S, and T waves at the same time when detecting R waves, and use the positions of these auxiliary waves to adjust the R waves detection. If the detection of these auxiliary waves is inaccurate, the accuracy of the R wave confirmation will also be affected.

**Figure 11:**
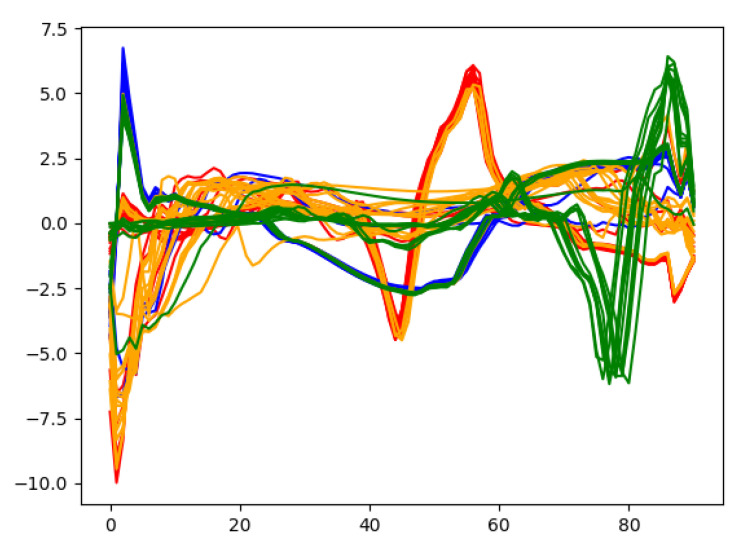
Some features extracted from the autoencoding network.

In order to gain deep insights on the learned heartbeat features, Figure 11 plots the extracted wearable ECG heartbeat features for subject no. 10 by the proposed multi-layer convolution autoencoding network, where one can clearly see that the features learned by autoencoding maintain discriminative features and provide a guarantee for accurate heartbeat separation.

In addition, we analyzed the effect of the sample length on the performance of heart rate estimation. Table 5 shows the average heart rate estimation error under different sample lengths of the ECG signals from five randomly selected subjects. We can see that, as the sample length increases, the heart rate estimation error tends to decrease first and then increase. Because when the sample length is very short, each sample cannot contain a complete heartbeat, which makes the algorithm unable to effectively extract the features of the heartbeat. The algorithm has the best performance when the sample length is set to 91 sampling points (0.91 s). Since the sampling frequency applied to the ECG signal is 100 Hz, setting the length of each sample to 91 sampling points (0.91 s) can make almost every sample contain a full heartbeat cycle, which will be more conducive to the extraction of the ECG signal R wave features.

We also investigated the effects of different depths of autoencoder layers on the performance of heart rate estimation. Table 6 shows the average heart rate estimation error when the number of convolutional layers of the autoencoder was set to 3, 6, 9 on the ECG data of five randomly picked subjects. It can be seen from Table 6 that when the number of convolutional layers was 3, there was an under-fitting phenomenon in the autoencoder training, which led to a larger heart rate estimation error. When the number of convolutional layers was 9, the heart rate estimation performance significantly improved compared to the 3-layer setting, but it was almost the same as the number of convolutional layers as 6. This shows that when the number of convolutional layers is small, increasing the number of convolutional layers can effectively improve the estimation performance. However, when the number of convolutional layers increases to a certain level, as the number of convolutional layers increases, the improvement in estimation performance will become insignificant. And it needs to be noted that the increase in the number of convolutional layers will lead to increased computational complexity of model training, reduce the time efficiency of model training, and increase the risk of the model overfitting.

## 5. Conclusions

This paper introduces the adaptive mechanism of the number of clusters in the clustering, and automatically selects the optimal number of clusters by setting the clustering evaluation function, which also ensures the high accuracy of the final heartbeat detection.

In this paper, the deep autoencoding strategy is introduced into the feature extraction of ECG wearable sensor signals, and a heartbeat detection and beat-to-beat heart rate estimation method is proposed based on convolutional autoencoding and Gaussian mixture clustering. By training convolutional autoencoding network, the high-level R wave features are extracted in an unsupervised manner, which could be applied for Gaussian mixture clustering for heartbeat confirmation. Experimental results show that the proposed method has achieved superior performance with an average mean absolute error as 0.67 beat/min, providing a robust beat-to-beat heart rate estimation solution for wearable ECG systems in practice.

## Figures and Tables

**Table 2:** The signal length of subject data.

Subject	Signal Length(s)
1	360
2	570
3, 5	420
4	540
6–10	180

**Table 3:** The comparison algorithms of this paper.

Method Abbreviation	Method
PT	Pan–Tompkins [13]
AMPD	Automatic peak detection [24]
METO	Multilevel Teager energy operator [23]
UNSW	UNSW [25]
HT	Hilbert transform [37]
Evo-MIACE	Evolutionary optimized multiple instance concept learning [12]
XGBoost	eXtreme gradient boosting [36]

**Table 4:** Performance of AG-GMM and comparisons across the 10 subjects, bold for the best, underline for the second best, standard deviations smaller than 0.01 are denoted as 0.00.

Subject	Mean Absolute Error (beat/min)							
	PT [13]	AMPD [24]	METO [23]	UNSW [25]	HT [37]	Evo-MIACE [12]	XGBoost [36]	AE-GMM
1	2.27	0.09	16.16	22.32	3.45	2.28 ± 0.15	2.24	**0.05**
2	1.00	1.55	**0.92**	1.18	3.53	2.19 ± 0.59	2.93	1.07
3	0.15	0.17	0.04	24.65	1.65	0.92 ± 0.50	1.30	**0.00**
4	0.09	0.16	**0.00**	0.05	2.67	1.25 ± 1.34	2.18	0.19
5	0.58	0.58	0.62	**0.42**	1.54	3.29 ± 0.70	2.47	0.96
6	**0.45**	20.02	0.80	2.56	1.36	1.59 ± 0.10	2.51	1.58
7	2.91	20.18	2.56	6.50	**0.73**	2.31 ± 0.29	1.63	1.48
8	**0.00**	0.71	0.76	0.00	3.71	3.76 ± 1.22	1.95	0.13
9	**0.00**	1.77	**0.00**	0.56	2.07	6.27 ± 2.64	3.49	0.91
10	12.64	0.28	0.30	0.12	2.91	**0.00 ± 0.00**	1.64	0.36
Total average	2.01	4.55	2.22	5.84	2.36	2.39	2.23	**0.67**

**Table 5:** Heart rate estimation error under different sample lengths. The bold are the sample length set in our method and the heart rate estimation error.

**Sample length**	31	61	121	151	**91**
**Average error**	2.49	2.02	1.31	1.29	**0.51**

**Table 6:** Heart rate estimation error under different number of convolutional layers of autoencoder. The bold are the number of layers set in our method and the heart rate estimation error.

**Number of layers**	3	9	**6**
**Average error**	1.01	0.54	**0.50**
